# Role of microRNAs in embryo implantation

**DOI:** 10.1186/s12958-017-0309-7

**Published:** 2017-11-21

**Authors:** Jingjie Liang, Shaoyu Wang, Zhengguang Wang

**Affiliations:** College of Animal Sciences, Zhejiang University, 866 Yuhangtang Road, Hangzhou, 310058 People’s Republic of China

**Keywords:** Implantation, Viable embryo, Endometrial receptivity, MicroRNA, Extracellular vesicle

## Abstract

Failure of embryo implantation is a major limiting factor in early pregnancy and assisted reproduction. Determinants of implantation include the embryo viability, the endometrial receptivity, and embryo-maternal interactions. Multiple molecules are involved in the regulation of implantation, but their specific regulatory mechanisms remain unclear. MicroRNA (miRNA), functioning as the transcriptional regulator of gene expression, has been widely reported to be involved in embryo implantation. Recent studies reveal that miRNAs not only act inside the cells, but also can be released by cells into the extracellular environment through multiple packaging forms, facilitating intercellular communication and providing indicative information associated with physiological and pathological conditions. The discovery of extracellular miRNAs shed new light on implantation studies. MiRNAs provide new mechanisms for embryo-maternal communication. Moreover, they may serve as non-invasive biomarkers for embryo selection and assessment of endometrial receptivity in assisted reproduction, which improves the accuracy of evaluation while reducing the mechanical damage to the tissue. In this review, we discuss the involvement of miRNAs in embryo implantation from several aspects, focusing on the role of extracellular miRNAs and their potential applications in assisted reproductive technologies (ART) to promote fertility efficiency.

## Background

Embryo implantation is a crucial step of pregnancy establishment in mammals and occurs in a restricted time period, termed the “window of implantation” (WOI). During implantation, the embryo and the uterus go through synchronous development and bidirectional crosstalk, eventually establishing structural linkage and achieving material exchange.

Understanding the mechanism of implantation has a profound effect on improving reproductive efficiency. Efficiency of pregnancy in human remains relatively low (~30%), and implantation failure accounts for 75% of pregnancy loss [1]. Situation in animals is slightly more optimistic, however, embryo loss during the pre-implantation period, which is very likely to happen in pigs and horses, remains a major obstacle to successful pregnancies [2]. Also in dairy cows, early embryo loss due to the failure of maternal recognition of pregnancy is believed to account for up to 25% of failures of conception [3]. Although ART has brought solutions to some fertility problems, implantation rate has not been greatly improved, and challenges remain regarding the poor accuracy of the methods to assess embryonic viability and endometrial receptivity. Thus, more investigations are needed in order to provide practical solutions to these problems.

Strategies for implantation vary considerably among species (Table 1) [2, 4, 5].Depending on the extent of the interactions between embryonic tissue and the maternal uterus, implantation can be invasive or non-invasive [4]. Primates and rodents exhibit invasive implantation, where the trophoblast cells of the blastocyst intrude into the uterine epithelium, penetrate the basal lamina, and form hemochorial placentation. Some domestic animals such as ruminants, horses, and pigs present non-invasive implantation, where the embryonic cells remain superficial or slightly fuse with the endometrial epithelium cells (EEC), forming synepitheliochorial (ruminants) or epitheliochorial placenta (pigs and horses). However, the initial stages of implantation are common across these species and are known as the “adhesion cascade for implantation” [4]. This process involves apposition and adhesion of the hatched blastocyst to the uterine luminal epithelium. Besides, embryo viability, endometrial receptivity and embryo-maternal crosstalk are the determinants for a successful implantation despite the difference in mammalian implantation strategies [6]. Moreover, the process of implantation is under the strict regulation of ovarian hormones- estrogen and progesterone [7]. Multiple molecules such as cytokines, chemokines, growth factors, lipids, and receptors also participate in the regulation of implantation through autocrine, paracrine and juxtacrine ways [7].

**Table 1:** Embryo implantation in different species

Species	Arrival to the Uterine Cavity	Hatch	Conceptus Elongation	Recognition Signal of Pregnancy	Initiate Implantation	Firm Attachment	Placentation
*Homo sapiens*	Day 4	Day 4–5	No	Human chorionic gonadotropin (hCG)	Day 6–7	Day 8–10	Hemochorial (invasive)
*Mus musculus*	Day 3	Day 4	No	Prolactin (PRL)	Day 4	Day 5–6	Hemochorial (invasive)
*Bos taurus*	Day 4–5	Day 9–10	Yes	Interferon tau (IFNT)	Day 19	Day 40–45	Synepitheliochorial (non-invasive)
*Sus scrofa*	Day 2–2.5	Day 6	Yes	Estrogen	Day 12–13	Day 25–26	Epitheliochorial (non-invasive)
*Ovis aries*	Day 3–4	Day 7–8	Yes	Interferon tau (IFNT)	Day 14–15	Day 28–35	Synepitheliochorial (non-invasive)
*Equus caballus*	Day 6	Day 7–8	No	Unknown factor	Day 35–40	Day 95–105	Epitheliochorial (non-invasive)

MiRNAs are small non-coding RNAs functioning in RNA silencing and post-transcriptional regulation of gene expression [8, 9]. Recent studies show that miRNAs are expressed in blood plasma and serum [10], as well as other body fluids [11]. Nearly all types of cells are able to secrete miRNAs and the concentration of extracellular miRNAs is considered to be associated with physiological and pathological conditions of the body [12]. Some extracellular miRNAs may also be implicated in intercellular communication [13, 14].

The process of implantation involves a complex regulation system that coordinates the embryo and maternal uterus. Evidence of miRNAs regulating embryonic development and uterine functions during the peri-implantation period suggests an important role of miRNAs in this process. Moreover, the discovery of extracellular miRNAs in uterine luminal fluid (ULF) as well as in embryo culture media prompts the need to explore novel potentials of miRNAs, especially in assisted reproduction. Based on their conservativeness, stability, sensitivity and easy access, extracellular miRNAs are suggested to be valuable non-invasive biomarkers for the assessment of embryo viability and endometrial receptivity. This review summarizes available information among species and discusses the impact of miRNAs, especially extracellular miRNAs, on the process of implantation from the perspectives of key factors that influence the implantation outcomes.

## Biogenesis and secretion of miRNAs

MiRNAs are small non-coding RNAs of length ~22 nt, regulating gene expression on the post-transcriptional level by targeting mRNAs for cleavage or transcriptional repression [8, 9]. Synthesized in the nucleus, a long and stem-loop carried primary miRNA is processed by Dorsha, a critical RNase III protein, to form a small hairpin-shaped miRNA, termed a pre-miRNA. Later, the pre-miRNA is translocated by protein exportin 5 (XPO5) from the nuclear pore complex to the cytoplasm, where it is cleaved by another critical RNase III protein, Dicer, into a small RNA duplex. Subsequently, the strand with a relatively unstable terminus at the 5ʹ side (in most cases) of the duplex is chosen as the guide strand to be loaded onto the Argonaute (AGO) protein family to form an effecter complex referred to as the RNA-induced silencing complex (RISC). It is the RISC that executes the function of RNA silencing or translational repression. Widely existing in cells and tissues, miRNAs are involved in a variety of biological processes. Multiple studies present the role of miRNAs in female reproduction [15]. They are involved in the regulation of oogenesis, fertilization, implantation, and placentation. Dysregulation of miRNAs has been shown to be associated with reproductive disorders, such as polycystic ovarian syndrome [15], and endometriosis [16].

Recent studies revealed that miRNAs can also be secreted into the extracellular environment. MiRNAs have been found in peripheral blood [10, 17] as well as other bio-fluids such as breast milk [18], saliva [19], urine [20], semen [21], and ULF [22]. These extracellular miRNAs stay in stable forms and are protected from endogenous RNases, showing feasibility of being non-invasive biomarkers for detection and diagnosis of pathological conditions, including cancers.

**Fig. 1:**
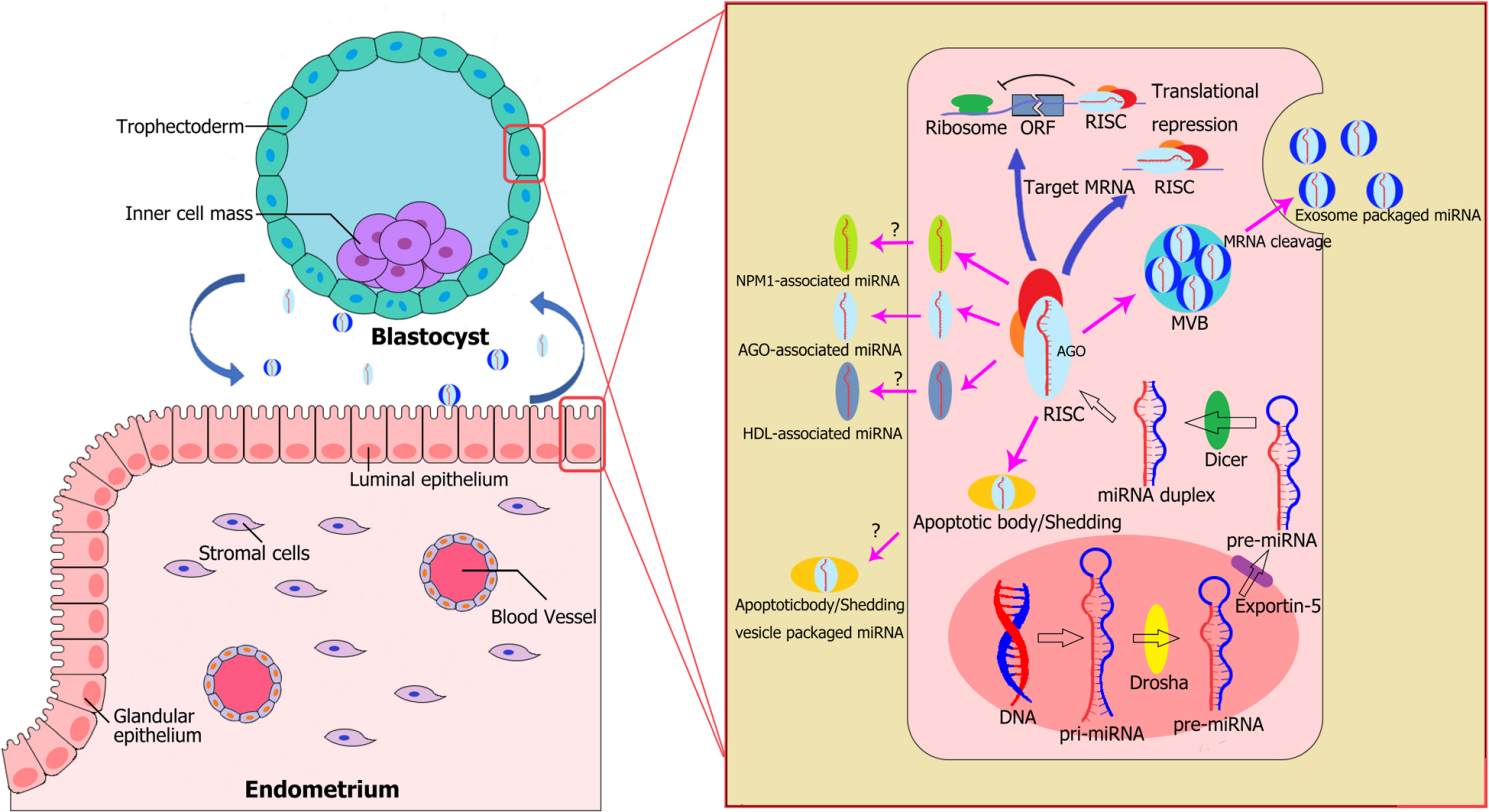
Extracellular miRNAs participant in embryo-maternal interactions. MicroRNAs (miRNAs) are synthesised in the nucleus as primary miRNA (pri-miRNA) transcripts. Pri-miRNAs are then processed by Drosha to form nucleotide hairpin known as miRNA precursors (pre-miRNA). Later pre-miRNAs are transported by exportin-5 from nucleus into the cytoplasm where they are cleaved by Dicer into small RNA duplex. Eventually, the guide miRNA strand is loaded onto Argonaute (AGO) protein family to form the RNA-induced silencing complex (RISC),leading to either mRNA cleavage or translational repression. MiRNAs can be secreted by cells through multiple forms: associated with proteins (e.g. Argonaute family or AGO, nucleophosmin 1), bound to lipoproteins (High-density lipoprotein), encapsulated into apoptotic bodies or membrane-bound extracellular vesicles (EVs). Uterine- and embryo-derived miRNAs have been reported to be associated with exosomes and AGO1, it remains unclear whether other forms of packaging are applied by endometrial cells and/or embryonic cells (question marks)

The main forms of extracellular miRNAs are associated with proteins (e.g. AGO family [23], nucleophosmin 1 [24]), bound to lipoproteins (high-density lipoprotein [25]), encapsulated into apoptotic bodies [26], or membrane-bound extracellular vesicles (EVs) [27] (Fig. 1). Among these packaging forms, protein-bound miRNAs take up the largest proportion of extracellular miRNAs in cell-free plasma and cell culture medium [28]. Though the concentration of EV-associated miRNA is far lower compared with other extracellular miRNAs, it is the most widely proved form of miRNA that can be selectively enriched, actively released by cells, and exert biological functions [29].

EVs are membrane vesicles released by cells and serve as vehicles transporting lipid, proteins and nucleic acids, including miRNAs [30]. According to their size and secretory manner, EVs can be divided into microvesicles (100-1000 nm in diameter) and exosomes (smaller than 150 nm in diameter). All EVs have specific molecules on the surface enabling them to be targeted to the recipient cells. Once they reach their recipient cells, EVs would release the cargos through binding to the receptors on the membrane surface, or endocytosis, or directly fuse with the membrane [31], and modify the functions of the recipient cell. The cargos of EVs are not randomly equipped. Many studies have shown selective packaging and enrichment of individual proteins and RNAs in secreted EVs [32], suggesting that the release of specific EVs is closely related to body conditions. However, the mechanism of selective packaging is not well understood. It has been studied that EVs participate in multiple physiological and pathological processes. There is an enthusiastic interest in investigating the role of EVs in tumor genesis, invasiveness and metastasis [33, 34]. Cancer-derived EVs were shown to modify the invasive ability of the tumor cells and promote dissemination. Moreover, EVs released by tumors can act on surrounding and distant non-tumor cells to assist in creating an optimal microenvironment for tumor growth [35]. It is worth noting the common features present between the behavior of invasive embryonic cells and that of cancer cells. Similar cellular mechanisms of cell adhesion, migration, invasion, and angiogenesis are shared during implantation and cancer spread [36]. As exciting reports springing up in the field of cancer study, it is reasonable to speculate a possible and impressive role that EVs and their cargos, especially miRNAs, may play in embryo implantation [37].

## MiRNAs participate in embryo implantation

### MiRNAs and embryo

#### Intracellular miRNAs influence embryo viability

Embryo viability is one of the key factors affecting implantation. During their development from zygotes to blastocysts, mammalian embryos undergo multiple events, including cell division, proliferation, establishment of cell polarity, compaction and lineage differentiation [38]. Unlike primates and rodents, whose embryos almost immediately attach to the endometrium after shedding from the zona pellucida, the embryo of ruminants and pigs experience a process termed ‘elongation’ where the embryo goes through morphological changes, developing from a spherical shape to an oval or tubular shape, and eventually to a filamentous form before attachment [4]. In horses, the embryo undergoes an extended period of mobility, growing as an ovoid shaped conceptus without obvious morphological extension [39].

Most of the embryonic changes can be related to activity of the genome [40]. Studies in mice revealed that the genomic information within pre-implantation embryo experiences wave-like changes associated with degradation of maternal transcriptome and zygote genome activation (ZGA) [41]. Though miRNA functions as a transcriptional regulator, its role in clearance of the maternal genome remains controversial. Studies have shown that miRNA function is generally suppressed in mouse oocytes and early pre-implantation embryos [42]. MiRNA activity was inhibited prior to the 2-cell stage, which is consistent with the timing of large-scale maternal gene degradation. However, the suppression is relieved after 2-cell stage and the expression of miRNA is reactivated [43].

Despite the repression of miRNA activity during ZGA, deletion of zygotic Dgcr8, which encodes an RNA-binding protein specifically required for miRNA processing, results in embryonic arrest prior to E6.5 [42]. Knocking out other key enzymes in the miRNA biosynthesis pathway such as DICER [44] and AGO2 [45] also leads to embryonic death around gastrulation, suggesting an important role of miRNA in early embryonic development [46]. Analysis of embryos at different developing stages revealed variable trends in miRNA expression, and the role of specific miRNAs in embryonic development has been studied in multiple species. MiR-29b might contribute to disruption of DNA methylation by regulating the expression of *DNMT3a/b*, which leads to early embryonic developmental blockade in mice [47]. Higher expression level of miR-130b was verified in the morula and blastocyst stages of bovine IVF produced embryos, while inhibition of this miRNA significantly reduced morula and blastocyst formation [48]. By inducing embryonic stem cells to differentiate into trophectodermal cells, miR-297, miR-96, miR-21, miR-29c, let-7, miR-214, miR-125a, miR-424 and miR-376a were suggested to be involved in trophectoderm specification [49]. MiR-519d, miR-378a-5p, miR-376, and miR-155 were reported to regulate the migration and invasion ability of human trophoblast cells [50–53]. MiRNAs are also implicated in regulation of embryo elongation. Functional annotations for comparisons among porcine conceptuses collected on Day10 (spherical/ovoid shape), Day 12 (filamentous form), Day 16 (elongated shape), and Day 20 (presence of evident vascularization on embryonic tissues) revealed that the differently expressed miRNAs were associated with cell cycle, cellular development, tissue morphology, inflammatory response and organismal development [54].

Environmental factors can regulate embryo viability by altering the expression of miRNAs. The mice embryo, when exposed to a progesterone-primed uterus, becomes metabolically dormant, and implantation is delayed. However, dormant embryos can be rapidly activated by a slight stimulus of estrogen and regain their implantation competency [7]. Delayed implantation in mice can be artificially induced through progesterone injection, which provides an excellent model for investigating the environmental influence on embryo implantation. Liu et al. [55] examined the miRNA profiles between dormant and activated mouse embryos and found that 45 miRNAs were differently expressed. Five of the let-7 family were down-regulated after activation. Further investigation revealed elevated let-7a reduced the number of implantation sites partly through targeting integrin beta 3. Another study in mice verified the relatively high level of let-7a in dormant embryos and found that this miRNA could also inhibit the expression of Dicer and prevent embryo implantation [56]. Although there is no evidence that delayed implantation exists in human and domestic animals, studies in mice suggest that in vivo environmental signals alter the miRNA expression patterns and eventually influence the active status of the pre-implantation embryo.

An embryo can be produced in vitro. However, production methods affect the expression pattern of miRNA in pre-implantation embryos, which in turn, affect embryo viability. In vivo fertilized porcine embryos presented lower expression of miR-24 in the blastocyst stage compared with in vitro fertilized (IVF) embryos [57]. A study in cattle revealed that elevated expression of miR-24 inhibited the development of embryo to the blastocyst stage [58]. Considering miR-24 is highly conserved across mammalian species, it may serve as biomarker for embryo quality. Down-regulated miR-199a-5p was shown in IVF mouse embryos compared with in vivo produced embryos, leading to higher glycolytic rate and lower developmental potential of blastocyst as well as reduced number of survived fetuses [59]. Together, these results reinforce the epigenetic modifications induced by the environmental factors on embryo development, since in vitro environment does not perfectly capitulate normal environment in maternal uterus [59], clarifying the functional miRNAs may help to improve the IVF system by artificially adjusting the amount of specific miRNA expression.

#### Extracellular miRNA profiles reflect embryo viability in vitro

Recent studies demonstrate that miRNAs exist not only within the embryo but can also be secreted by the embryo to the extracellular environment. MiRNAs have been detected in the culture media (CM) derived from IVF human and bovine embryos, with their unique expression profiles associated with the embryonic developmental and chromosomal status, sexual dimorphism, and the reproductive competence after transfer to the uterus.

Kropp et al. [60] compared the expression of several miRNAs between IVF bovine blastocysts and degenerate embryos (IVF embryos failed to develop to the blastocyst stage) and found relatively higher levels of miR-181a2, miR-196a2, miR-302c and miR-25 in degenerate embryos. They further investigated the miRNA contents in the CM and found that miR-25 was only present in CM containing embryos but not in the control media (embryo free). This finding suggested that miRNAs might be secreted by the embryos. Additionally, the absence of miR-302 in all media indicated that the secretion of miRNAs was selective. In another study, Kropp and Khatib [58] applied deep sequencing to characterize miRNA profiles in CM from IVF produced bovine blastocysts and degenerate embryos, and miR-24 was confirmed to be highly expressed in CM from degenerate embryos. Addition of miR-24 mimics to the CM from normal morula significantly reduced the development rate of embryos, partly through inhibiting cell proliferation by targeting *CDKN1b*, a cell cycle inhibitor. The result indicates the significant effect of extracellular miRNAs on embryo development.

The presence of miRNAs has also been confirmed in the CM from IVF human embryos. Rosenbluth et al. [61] found that miR-645 was only expressed in the control media and was found to be undetectable in CM from embryos. On the contrary, miR-372 and miR-191 were only detected in the spent CM. Moreover, the expressions of extracellular miR-372 and miR-191 were associated with IVF failure, since they were both highly detected in CM from failed IVF cycle embryos. Interestingly, a relatively higher level of miR-191 was discovered in CM from aneuploid embryos, indicating the possible role of miR-191 as a biomarker of embryo aneuploidy, a major cause of recurrent implantation failure.

Capalbo et al. [62] conducted a comprehensive analysis of miRNA profiles between the spent blastocyst culture media (SBCM) collected from human euploid blastocyst that failed to implant and blastocyst that implanted. Two miRNAs, miR-20a and miR-30c, presented significantly higher expression in SBCM from implanted blastocysts. Intriguingly, the target genes (such as *PTEN, NRAS, MAPK1, APC, KRAS, PIK3CD, SOS1*) of these miRNAs were predicted to be involved in endometrial cell proliferation, which suggested the potential of blastocyst secreting miRNAs as modulators of the uterine functions. However, this speculation was not verified in this study. The group also tested the CM from embryos at other developmental stages (cleavage and morula) and discovered that the analyzed miRNAs in SBCM were specific to the blastocyst stage, strengthening the point that during this particular stage the embryo may send signals to the environment in order to facilitate the subsequent implantation process.

A very recent study showed that embryos with different genders secreted different miRNAs [63]. A relatively abundant amount of miR-22, miR-122 and miR-320a were detected in CM from female bovine embryos. Taking into account that male and female embryo apply different adaptations to the external environment, they may secrete different miRNAs into the maternal environment, inducing transcriptional response of the mother to create an appropriate environment for their development.

The detection of miRNAs in the CM from pre-implantation embryos shed new light for embryo screening in the IVF process. At present, the methods used for screening embryos can be categorized as non-invasive ways and invasive ways. Non-invasive screening is mainly based on the morphological observation and metabolic profiling of the CM in order to determine the development status of embryos [64]. Although advanced technologies such as the time-lapse system have permitted keeping track of the development steps of an embryo under minimum variation of the culture environment [65], chromosomal abnormalities - which contribute to repeated failure of implantation, taking up 44.9% of morphological normal embryos [66] - cannot be ruled out. Invasive screening methods are able to identify the genomic information within the embryos. Novel technologies such as comparative genomic hybridization overcome the limitation of pre-implantation genetic screening and fluorescence in situ hybridization, promising a comprehensive analysis of chromosomes. However, the challenge of invasive screening remains regarding the damage to the embryos, and there is no definite conclusion that biopsy procedure will do no harm to the further development of embryos after they have been transferred to the uterus.

An ideal screening method should be non-invasive and accurate. Based on such consideration, miRNAs secreted by pre-implantation embryos may serve as potential biomarkers in the screening process because of their embryonic specificity, stability and easy access. However, how the secreted miRNAs are packaged might influence the judgement of embryo quality. Only one study has shown that embryonic secretion of miRNAs was carried through AGO1 [23]. None of the studies mentioned above have tested the exact release form of these extracellular miRNAs. What should be noticed is that the composition of the CM (e.g. the addition of BSA [60]) and the manipulation strategies (e.g. fertilization methods) [61, 67] have certain influence on extracellular miRNA profiles. Further investigation should clarify the forms of extracellular miRNAs within the CM in order to improve the accuracy of selection. Moreover, whether the discussed miRNAs can be used to reflect embryo viability is defined by their relative expression levels, rather than their existence, even though the latter makes them more ideal indicators. Hence, repeated experiments are required to establish measurement standards of miRNA expressions before taking them as effective biomarkers.

### MiRNAs and endometrium

#### Intracellular miRNAs participate in uterine events during peri-implantation

Uterine sensitivity to implantation can be divided into three phases: pre-receptive phase, receptive phase, and refractory phase [38]. Implantation can only occur on the receptive phase, when the uterus is able to accept and accommodate the embryo. Collaboration of estrogen and progesterone directs the uterus into the receptive phase, accompanied by morphological and functional changes in the epithelium and the stroma [7]. Investigations of genes (such as *DROSHA, DGCR8, XPO5, DICER, AGO1–4*) related to miRNA synthesis and transport revealed vivid activities of miRNA production, variable miRNA expression profiles at different endometrial stages suggest the regulatory role of miRNAs in endometrial receptivity [68]. Hsa-miR-30b and hsa-miR-30d were found to be significantly upregulated and hsa-miR-494 to be downregulated in the receptive endometrium (LH + 7) compared with the pre-receptive endometrium (LH + 2) from healthy fertile women. The predicted target genes of these miRNAs were involved in cyclic remodeling of the endometrium, including endometrial maturation to the receptive state [69]. In mice, decreased expressions of miR-181 and miR-223-3p on Day 4 of pregnancy (WOI) were shown to be essential for initiating implantation [70, 71], since these miRNAs lowered the expression of LIF, a promising marker of implantation, and impeded implantation. Increased expression of miR-223-3p also reduced the formation of pinopodes, large apical protrusions that appear on the surface of epithelium and that might serve as the preferred attachment site for the embryos [71].

In preparation for embryo adhesion, the endometrial luminal epithelium must convert to an adhesion competent state to support the interactions with the embryo [5], this includes the alternations of anti-adhesive components on the endometrial luminal epithelium. Mucin 1 (*Muc1*) is an integral transmembrane mucin glycoprotein expressed on the apical surface of endometrial epithelia, acting as an inhibitor of embryo attachment. The expression of *Muc1* in mouse decreased significantly during the implantation window, which might be regulated by miR-199a, let-7a, and let-7b. These miRNAs presented an inverse trend in the receptive endometrium [72, 73]. Type-1 insulin-like growth factor receptor (*IGF1R*) is up-regulated in the endometrial epithelium during the receptive stage. This increase might contribute to an adhesive interaction at the cell surface. High level of miR-145 inhibits embryo attachment partly through regulating endometrial *IGF1R*. This may provide an explanation for repeated implantation failure (RIF), since elevation of miR-145 has been shown in the endometrium of RIF patients [74].

During the receptive stage, the endometrial epithelium exhibits epithelial-mesenchymal transition (EMT) features, becoming less polarized and displaying remodeling of cell junctions to facilitate interaction with trophectoderm [5]. As a member of the miR-200 family who play a critical role in the suppression of EMT [75], miR-429 exhibited a declined expression during implantation in mice. Enhancement of miR-429 resulted in suppression of the migratory and invasive capacities of cells probably through targeting protocadherin 8, leading to reduced implantation sites [76]. On the contrary, miR-126-3p was specifically up-regulated in implantation sites, promoting cell migratory and invasive capacity by regulating the expression of integrin α11 [77]. Progesterone induced the expression of miR-125b in human EEC. Increased miR-125b inhibited cell movement and impeded implantation by suppressing the expression of MMP26, a member of the matrix metalloproteinase family which is involved in degradation of extracellular matrix [78].

In primates and rodents with invasive implantation, penetration of the trophoblast triggers a series of stromal response termed decidualization, which involves massive proliferation, differentiation and apoptosis of the stromal cells [4]. Some miRNAs are enhanced during decidualization. Mmu-miR-96 promoted the apoptosis of stromal and decidual cells by regulating anti-apoptotic factor Bcl-2 [79]. MiR-181a stimulated the expression of human endometrial stromal cell (hESC) decidualization-related gene (such as *FOXO1A, PRL, IGFBP-1, DCN, TIMP3*) and induced morphological transformation [80]. Other miRNAs are repressed during this period. MiR-222 participated in ESC differentiation by regulating ESC terminally withdrawing from the cell cycle partly through permitting *CDKN1C/p57* [81]. Down-regulation of mmu-miR-200a and mmu-miR-141 facilitated the expression of *PTEN*, which in turn influenced cell proliferation and apoptosis during decidualization [82, 83].

#### Uterine luminal fluid exhibits specific extracellular miRNA profiles

Growing interests in extracellular miRNAs have led to the speculation of whether endometrium is able to secrete miRNAs. Studies have confirmed that miRNAs are encapsulated within EVs in ULF and uterine aspirates in human [84], sheep [22], and pigs [54]. Immunostaining results of membrane markers of exosomes in vivo highlighted an increased secretion trend in both luminal and glandular epithelial cells across the menstrual cycle, and the secretion reached a peak during WOI [85]. In vitro experiments compared the miRNA profiles in EEC with that in their secreted exosomes and found 13 among the 227 miRNAs were exclusively present in exosomes/microvesicles while five miRNAs were unique to EEC. The target genes of these exosome-enriched miRNAs were involved in several signaling pathways associated with implantation [86]. Another study in ewes revealed 53 commonly expressed miRNAs in ULF extracellular vesicles derived from both the cycling ewes and the pregnant ewes on Day 14, and one miRNA, bta-miR-423, was solely detected in the pregnant sample. Bta-miR-423 is thought to target genes associated with metabolism, immune system, cell cycle and apoptosis [22].

In the process of IVF, inadequate uterine receptivity is considered to be responsible for nearly 2/3 of implantation failures [87]. Current methods used for the endometrial receptivity evaluation is based on morphological assessment and genome investigation. Large number of molecules have been proposed as receptive biomarkers, however, these markers sometimes present differences among individuals which bring misleading judgments on the fertility status [88]. The detection of extracellular miRNAs in ULF bring new options for the non-invasive diagnosis of endometrial receptivity, but more investigations need to be involved before it become a real approach in clinical practice.

### MiRNAs in embryo-maternal interaction

Synchronized development between the embryo and the endometrium is essential for successful implantation, and embryo-endometrial asynchrony beyond a certain time period leads to declined implantation rate [89]. To ensure synchronization, conversation between embryo and the uterus must hold. Besides the regulation of estrogen and progesterone, both the embryo and the endometrium can secrete unique signals to inform the other party, adjusting the pace of their development. For example, the embryo releases pregnancy recognition signals (e.g. chorionic gonadotropin for human, interferon tau for ruminants) to prevent luteolysis by prostaglandin F2α in order to maintain a mild environment for pregnancy, while uterine secretions regulate the embryo development status and promote trophectoderm proliferation, migration, as well as attachment to the endometrial luminal epithelium.

There is a growing interest in studying the microenvironment where bidirectional communication takes place. Within the uterine cavity, ULF - secreted by luminal and glandular epithelia and in intimate contact with the embryo and endometrial epithelium - is considered to be essential for embryo development and implantation [90, 91]. The secretion profiles of the ULF are believed to reflect the receptive state of the endometrium, thus ULF has been proposed to serve as a source of non-invasion biomarkers.

MiRNAs, contained within exosomes/microvesicles, have been detected in ULF and uterine aspirates among species. It was suggested that they might have a role in embryo-maternal interactions during implantation (Fig. 1). Vilella et al. [85] demonstrated a variable expression pattern of miRNAs in human endometrial fluid secreted by the endometrial glands at different stages of the menstrual cycles. The group compared the WOI with the rest of the menstrual cycle and discovered that hsa-miR-30d was the most differently expressed miRNA. Further investigation revealed that this unique miRNA could be transported through exosomes. By using an in vitro mouse model, miR-30d carried through exosomes was shown to be internalized by mouse blastocysts and modified the embryonic transcriptome and phenotype. Supplement of mimic miR-30d to the embryo led to overexpression of ten genes (such as *ITGB3, ITGA7* and *CDH5*) which are related to cell adhesion, integrin-mediated signaling pathways and developmental maturation. The adhesion rate of embryo was also improved.

In another study, Cuman et al. [23] provided more direct evidence that miRNA secreted by the embryo participates in modulating uterine functions. CM from human blastocysts with opposite implantation outcomes were analyzed, and the concentration of miR-661 was reported to be significantly higher in the CM collected from blastocysts that failed to implant than those who implanted successfully. The group later used CM instead of supplement of miRNA to show that the enhanced level of miR-661 in the extracellular environment elevated the intracellular expression of miR-661 in cultured human EEC. Further investigation unraveled that miR-661 was transported by AGO1 and taken up by human EEC. Elevated miR-661 expression inhibited the attachment of trophoblast cell line spheroid to human EEC, partly via PVRL1, a membrane-bound immunoglobulin-like cell adhesion molecule.

These studies show that both the embryo and the uterus can secrete specific miRNAs according to their own conditions, and these secreted miRNAs are likely to be taken up by the other party to modify the transcriptomes in them to facilitate implantation.

## Circulating miRNAs and pregnancy

Circulating miRNAs refer to cell-free miRNAs which are present in peripheral blood [92]. Many studies show circulating miRNAs as promising biomarkers for the diagnosis and prognosis of several cancers [93]. There is certain evidence that circulating miRNAs have indicative functions in the process of pregnancy. Placenta-specific miRNAs, mainly linked to the chromosome 19 miRNA cluster, are widely expressed in the blood plasma of pregnant women [94, 95]. These miRNAs (e.g. miR-515-3p, miR-517a, miR-517c, miR-518b, miR-526b, and miR-323-3p) are probably released by the trophoblast cell into the maternal circulation system through exosomes during pregnancy, and their presence is rapidly cleared after parturition [96]. Other studies highlight that miRNAs are possible indicators for pregnancy failure and complications. Patients with ectopic pregnancy (EP) or spontaneous abortion (SA) were reported to carry a significantly lower serum concentration of miR-517a, miR-519d, and miR-525-3p compared with women with viable intrauterine pregnancy; a unique high expression of serum miR-323-3p was found in the EP group, which helps to distinguish EP and SA, showing potential of being a biomarker [97].

Only few studies aimed to investigate the correlation between circulating miRNAs and embryo implantation. Kresowik et al. [98] screened the expression of several miRNAs in the endometrial tissue and serum of fertile women and discovered that miR-31 was significantly up-regulated in serum during the WOI, which was consistent with its expression in tissue. In vitro experiments suggested that this remarkable increase was associated with the rise of progesterone level. Immune related genes such as *FOXP3* and *CXCL12* were significantly downregulated in the endometrial tissue due to the increase of miR-31, suggesting this miRNA plays a role in regulating the immune system during implantation. Ioannidis et al. [99] profiled plasma miRNAs from pregnant and non-pregnant heifers and discovered that the concentration of miR-26 was higher in pregnant heifers on Day 16 of pregnancy and the level of this miRNA increased from Day 16 to Day 24. Considering the onset of implantation in cattle is around Day 19 after fertilization [2], miR-26 might be a candidate biomarker for very early pregnancy in cows. Another study in cows revealed that circulating EV-derived miRNA is not only able to identify pregnancy, but is able to distinguish between successful implantation and embryonic mortality at the early stage of pregnancy [100]. The expression of miR-25, miR-16a/b, and miR-3596 at day 17 was higher in the embryo mortality group compared to both the pregnant and control groups, suggesting their potentials in differentiating pregnancy status. Differentially expressed miRNAs were also confirmed in serum exosomes collected from pregnant and non-pregnant mares, showing potential role in maternal recognition of pregnancy probably through regulating the focal adhesion pathway [101].

Circulating miRNAs have been suggested to be effective biomarkers due to their stability, informativeness and non-invasive detection. However, defining the existence form of circulating miRNAs is necessary. Many studies are conducted on EV-derived miRNAs, though this form of miRNA accounts for only a small fraction of the total extracellular miRNAs [29]. Whether other forms of extracellular miRNAs have indicative functions remains to be studied. Moreover, the result of circulating miRNAs studies can be affected by experimental and analytical method [102]. Further effort applying standardized and consistent method will be required to determine whether circulating miRNAs can be used as reliable biomarkers for implantation events, especially for detecting endometrial receptivity.

## Conclusions

Implantation is an elaborate process requiring the synchronous development of a viable embryo and a receptive endometrium. MiRNA works as a regulator of gene expression and is actively involved in regulating embryo development, endometrial functions, and embryo-maternal communications. The verification of functional extracellular miRNAs brings new opportunities for improving implantation outcomes mainly from two aspects: first, intercellular communication through extracellular miRNAs provides a new dimension for understanding the mechanism of implantation; second, extracellular miRNAs have the potential for being effective biomarkers in IVF-ET for detection and prognosis of embryo quality and endometrium receptivity.

In view of the pleiotropic effects of miRNAs, it is difficult to define the specific role of a particular miRNA. At present, most of the miRNA experiments are conducted on cellular level in vitro, but some groups also apply in vivo models by injecting miRNA mimics and (or) inhibitors. Indeed, the supplement of miRNA (or its inhibitor) will lead to expressional changes on the specific predicted target gene, which causes phenotypic changes in turn. However, it can not be excluded the possible effect of other potential target genes which might be regulated by the same miRNA. Since current experiments only provide limited potential of specific miRNAs, further investigations and more data are required to summarize the rule of miRNA regulation. In contrast, miRNAs, especially extracellular miRNAs, present a brighter future for developing non-invasive biomarkers. Besides the conservativeness, extracellular miRNAs are highly stable and sensitive in bio-environment. Meanwhile, they present specificity associated with physiological and pathological conditions. Non-invasive access is another striking advantage of extracellular miRNAs. However, it is necessary to define the package forms and secretion mechanisms of extracellular miRNAs, since this may influence our judgement on its function as well as the accuracy of evaluation when applying one as biomarker for certain conditions.

The exploration of relationship between extracellular miRNAs and implantation had only begun. More research should be done and their results should be replicated in clinical trials in order to bring true efficiency to implantation improvement.

## Abbreviations

AGO: Argonaute
ART: Assisted reproductive technology
CM: Culture media
EEC: Endometrial epithelium cells
EMT: Epithelial-mesenchymal transition
EP: Ectopic pregnancy
EV: Extracellular vesicles
FAM: Focal adhesion molecules
hESC: Human endometrial stromal cell
IGF1R: Type-1 insulin-like growth factor receptor
IVF: In vitro fertilization
RIF: Repeated implantation failure
RISC: RNA-induced silencing complex
SA: Spontaneous abortion
SBCM: Spent blastocyst culture media
ULF: Uterine luminal fluid
WOI: Window of implantation
XPO5: Exportin 5
ZGA: Zygote genome activation
